# Gp96 deficiency affects TLR4 functionality and impairs ERK and p38 phosphorylation

**DOI:** 10.1371/journal.pone.0193003

**Published:** 2018-02-15

**Authors:** Jesus Cosin-Roger, Marianne R. Spalinger, Pedro A. Ruiz, Claudia Stanzel, Anne Terhalle, Lutz Wolfram, Hassan Melhem, Kirstin Atrott, Silvia Lang, Isabelle Frey-Wagner, Michael Fried, Michael Scharl, Martin Hausmann, Gerhard Rogler

**Affiliations:** Department of Gastroenterology and Hepatology, University Hospital Zurich, University of Zurich, Zurich, Switzerland; Université de Genève, SWITZERLAND

## Abstract

Gp96 is an endoplasmic reticulum chaperone for multiple protein substrates. Its lack in intestinal macrophages of Crohn’s disease (CD) patients is correlated with loss of tolerance against the host gut flora. Gp96 has been stablished to be an essential chaperone for Toll-like receptors (TLRs). We studied the impact of gp96-knockdown on TLR-function in macrophages. TLR2 and TLR4 expression was only decreased but not abolished when gp96 was knocked-down in cell lines, whereas in a monocyte/macrophage specific knock-out mouse model (LysMCre) TLR4 was abolished, while TLR2 was still present. Lipopolysaccharide (LPS)-induced NF-κB activation was still observed in the absence of gp96, and gp96-deficient macrophages were able to up-regulate surface TLR4 upon LPS treatment, suggesting that there is another chaperone involved in the folding of TLR4 upon stress responses. Moreover, LPS-dependent pro-inflammatory cytokines were still expressed, although to a lesser extent in the absence of gp96, which reinforces the fact that gp96 is involved in regulating signaling cascades downstream of TLR4 are impaired upon loss of gp96. In addition, we have also found a reduced phosphorylation of ERK and p38 kinases and an impaired response upon CSF1R activation in gp96 deficient macrophages. Our findings indicate that the loss of gp96 not only impairs TLR4 signaling, but is also associated with a diminished phosphorylation of ERK and mitogen-activated stress kinases resulting in an impaired signalling through several receptors, including CSF1R.

## Introduction

The heat shock protein gp96, encoded by the *Hsp90b1* locus, is an endoplasmic reticulum (ER)-localized chaperone, whose significant role in innate as well as adaptive immunity was reviewed by Schild and Rammensee in detail, where its multifunctionality was compared with a pocket knife [1].

Gp96 can carry peptides, which are transferred to the MHC class I molecules of antigen presenting cells thereby mediating immunity against antigens from cells of its origin. On the other hand, gp96 also behaves like a cytokine by inducing and activating dendritic cells (DCs) [2] and triggering the expansion of CD8+ T cells [3].

Randow and Seed demonstrated the important role of gp96 for correct folding and export of Toll-like receptor (TLR) 1, TLR 2 and TLR 4 in mutant variants of a murine pre-B-cell line expressing a truncated gp96 [4]. Furthermore, gp96 has been suggested to be an important chaperone for TLRs in monocytes/macrophages [5, 6]. Lack of correct TLR protein folding is associated with impaired innate immunity. Yang et al. suggested that the function of gp96 in folding TLRs cannot be replaced by other chaperones [6]. In a mouse model of monocyte/macrophage specific gp96-deficiency a cell-specific loss of surface TLRs expression, which leads to a categorical loss of TLR function, was described. These mice were resistant to endotoxin shock, but were highly susceptible to *Listeria monocytogenes* and *Klebsiella pneumoniae* infection [6].

TLRs represent the best-investigated family of pattern recognition receptors (PRRs). These cell-activating receptors recognize bacteria and virus derived molecules or structures and are important elements of the innate immune system [7, 8]. TLRs induce a common signalling pathway leading to the activation of NF-κB as well as the mitogen-activated protein kinases (MAPKs), extracellular signal–regulated kinase (ERK), p38, and c-Jun N-terminal kinase (JNK) [9, 10]. Since the intestinal mucosa is challenged by a permanent contact with an indeterminable multiplicity of bacterial and food antigens from the intestinal lumen, intestinal macrophages (IMACs) act as a first barrier of the immune system in the gastrointestinal tract.

IMACs represent one of the largest macrophage populations of the human body [11–13]. In addition to initiating the first innate immune response at the site of the highest antigen density, IMACs mediate tolerance against food antigens and the commensal intestinal flora. This tolerogenic ability is mediated by a lack of the expression of typical macrophage activation receptors, including TLR2 and TLR4. IMACs are of crucial importance for pathogen recognition at the mucosal surface and an impairment of their innate immune functions has been associated with the pathogenesis of inflammatory bowel disease (IBD), in particular Crohn’s disease (CD). During IBD an increased immigration of monocytes into the intestinal mucosa can be observed[14–16]. These monocytes do not completely differentiate into mature, tolerogenic IMACs but maintain the expression of activation receptors on their cell surface [17] and strong expression of TLR2 and TLR4 is found on inflammation-associated IMACs [18].

Previous studies revealed a specific loss of gp96 protein in IMACs of CD patients but not in patients with diverticulitis or ulcerative colitis (UC). Of interest, mRNA levels of gp96 were unaltered in CD patients [19]. These findings suggest that a lack of gp96 in IMACs IN CD patients could be correlated with the loss of tolerance against the host gut flora, leading to chronic inflammation. Indeed, it is well known that changes in mucosal immunity and tolerance may impair mucosal barrier function leading to an increased penetration of microbial components and hereby triggering aberrant immune responses and inflammation [20].

We have previously reported a strong expression of TLR2 and TLR4 on inflammation-associated IMACs, leading to a higher susceptibility of CD patients to lipopolysaccharide (LPS) [18] as well as a specific loss of gp96 in IMACs of CD patients[19]. These findings are somewhat contradictory to the results from Yang *et al*. showing that gp96 acts as major chaperone for TLRs [6]. Therefore, in order to clarify these conflicting observations from CD patients, we studied the impact of gp96-knockdown on TLR-function in the human monocytic cell line Mono Mac 6 (MM6) and the relevance of this chaperone *in vivo*, using a macrophage-specific gp96 knock-out (KO) mouse that was generated for this project.

## Material and methods

### Material

All cell culture reagents were obtained from Invitrogen (Carlsbad, CA, USA), OPI media supplement and LPS from *E*. *coli* (0111:B4) were purchased from Sigma Aldrich (St. Louis, MA, USA). For Western blot analysis, cells were lysed in Pierce M-PER lysis buffer (Rockford, IL, USA) and applied on SDS-Gels with 4x NuPAGE sample buffer (Invitrogen). Nuclear fractions were gained by applying the Nuclear Extract Kit provided by Active Motif (Carlsbad, CA, USA) according to the manufacturer’s protocol. Antibodies against p-IκBα, IκBα, p-NF-κB, NF-κB, p-ERK, ERK, p-p38 as well as p38 were from Cell Signalling (Danvers, MA, USA), whereas the antibody against gp96 was purchased from Stressgen (Ann Arbor, Michigan, USA). The antibodies against β-actin and lamin A/C were from Millipore (Bedford, MA, USA) and BD Biosciences (Allschwil, Switzerland), respectively. Horseradish-peroxidase coupled secondary antibodies were from Santa Cruz. Chemiluminescent substrate ECL plus and films were from GE Healthcare (Chalfont, UK). IL-8 ELISA was obtained from Invitrogen. All other reagents were of analytical grade and acquired commercially.

### Mice

Mice were kept under specific pathogen-free conditions in the animal facility of the University Hospital Zurich with access to food and water *ad libitum*. All animal experiments were performed in accordance with institutional and state guidelines under animal experiment license No. ZH214/2014 (25719), approved by the Cantonal veterinary office of Zurich. TLR4^-/-^ and TLR2^-/-^ mice in the C57BL/6 background were a kind gift from Prof. M. Manz (University Hospital Zürich, Zürich, Switzerland).

### Generation of the LysMcre-gp96-flox mice

Embryonic stem (ES) cell clones were derived from EUCOMM (JM8A3N1 cell line, target locus: Hsp90b1). Three different clones were tested by Southern blotting using an internal probe in the neo sequence and long PCRs across the homology arms with one generic primer within the targeting cassette and locus specific primer outside the targeting vector. Clone Hsp90b1_G07 was used for blastocyst injection. Two male chimeras were obtained and crossed with albino females (B6(Cg)-Tyr-2J/J) to obtain mice that were crossed with transgenic FLP mice in order to remove the gene-trap cassette. Subsequently, mice were inbred to generate homozygous gp96-flox mice. Finally, LysMcre-g96-flox mice were obtained by crossing with LysMCre mice. All the pups were genotyped by two different PCRs using specific primers for the presence of the LysMCre recombinase 5’-cccagaaatgccagattacg-3’ and 5’-cttgggctgccagaatttctc-3’ and for the presence of gp96 locus 5’-gcctgaaagtcacactcaacttcc-3’ and 5’-ctaccctacagtctatgttatggc-3’. In both cases, PCRs were performed with the Qiagen Hot StarTaq Master Mix kit.

### Isolation of peritoneal macrophages

After sacrificing the mice by cervical dislocation, 10 ml of cold DMEM was injected intraperitoneally into WT and LysMCre-g96-flox mice and peritoneal exudate cells (PEC) were collected after holding and shaking the mice during 30 seconds. PEC were centrifuged for 10 minutes at 400g at 4°C, the supernatant was discarded and cells were resuspended in DMEM supplemented with FCS 10%. Approximately 2 million of cells were obtained per mice and all the cells were seeded in 6-well plates and incubated for 2 hours at 37°C in order to allow the adherence of macrophages. Subsequently, non-adherent cells were removed washing three times with PBS, and macrophages, the adherent cells, were cultured with DMEM supplemented with FCS 10% during the experiments.

### Cell culture

The monocytic cell line Mono Mac-6 (MM6) was maintained in RPMI medium containing 10% FCS, 1% OPI, 1% penicillin/streptomycin, 1% non-essential amino acids and 0.5% natrium pyruvate in a humidified atmosphere with 5% CO_2_ and cell counts were kept below 1x10^6^ cells/ml.

### Lentiviral knockdown

For lentiviral production the packaging plasmid pMD8.91, vesicular stomatitis virus (VSV), envelope-expression plasmid (pMD.G) and pLKO.1-puro gp96-knockdown vector (NM_003299.1-1816s1c1) or pLKO.1-puro mock-vector (both from Sigma Aldrich) were used. HEK 293T cells were cultured in a humidified atmosphere with 10% CO_2_ in 4.5% high glucose Dulbecco’s modified eagle medium (Invitrogen) supplemented with 10% newborn calf serum. Lentiviral vector particles were produced by co-transfection of HEK 293T cells with transfer, packaging, and envelope plasmids (2.5 μg, 1.5 μg, and 1.5 μg / 9.5 cm^2^), respectively using Fugene HD Transfection Kit (Roche, Basel, Switzerland). The virus containing supernatant was harvested 48 and 72 h later and sterile filtered through 0.20-μm-pore cellulose acetate filters (Sartorius Stedim Biotech, Goettingen, Germany). Transduction of MM6 cells (5x10^5^cells/ml) was carried out in the presence of polybrene (4 μg/ml; Sigma Aldrich). Stably transduced cells were obtained by puromycin selection (5 μg/ml). Puromycin-containing medium was renewed every 2–3 days. Stably transduced cells were used for experiments not earlier than 21 days after transduction.

### Stimulation of MM6 cells and peritoneal macrophages with LPS and M-CSF

LPS was re-suspended in ultrapure water to a concentration of 5 mg/ml. Further dilution to working concentration of 100 ng/ml was performed immediately prior to stimulation. Cell number was adjusted to 5x10^5^cells/ml. Cells were stimulated at different times (0, 30 min, 60 min, 120 min and 24 hours). M-CSF was resuspended in ultrapure water to a concentration of 0.1mg/ml. Peritoneal macrophages were treated with M-CSF 10 ng/ml for 3 days, and, then restimulated with M-CSF 10ng/ml for 10 minutes.

### Flow cytometry and cell sorting

Expression of TLR 2 and 4 on the cell surface was investigated by flow cytometry. MM6 cells were first stained with LIVE/DEAD Fixable Aqua Dead Cell Stain Kit (Life Technologies, Eugene, Oregon, USA) according to manufacturer’s instructions in order to exclude dead cells from the analysis. Cells were separately incubated with TLR2-PE antibody (eBioscience, San Diego, CA, USA) or TLR4 antibody (Novus Biologicals, Littelton, CO, USA) for 30 minutes on ice. After washing with FACS buffer, cells incubated with TLR4 antibody were incubated with the secondary antibody goat anti-mouse PE (Caltag Laboratories, Burlingame, CA, USA) 30 minutes on ice. In the case of peritoneal macrophages, cells were incubated with Fc Receptor blocking Reagent (Miltenyi Biotec) for 20 minutes. Subsequently, cells were washed with PBS and stained with Fitc-conjugated anti-F4/80 (eBioscience), BV605-conjugated anti-CD64 (BioLegend), PE-conjugated anti TLR2 or anti TLR4 (both from eBioscience), and Zombie NIR^™^ live death discriminator (BioLegend) for 15 minutes. Cells were washed with PBS, fixed with Fixation/Permeabilization buffer (eBioscience) for 5 minutes and incubated with unlabelled rabbit anti-phopho-NFkB p65 antibody (cell signalling) for 15 minutes. After washing with permeabilization/washing solution (eBioscience), APC-labelled anti-rabbit secondary antibody (eBioscience) was added for 15 minutes. Cells were washed twice with FACS buffer prior to analysis. All incubations were performed on ice. Cells were acquired on a FACS Canto II or LSR Fortessa Cytometer and compensation settings were defined using anti-rat/hamster Ig, k/ Negative Ctrl Compensation Set (BD Biosciences, Allschwil, Switzerland, Europe).

For cell sorting, macrophages were treated for 2 hours with LPS, washed 2 times with PBS and detached with ice cold EDTA (2mM) in PBS. Cells were then stained with Fitc-conjugated anti-F4/80 (eBioscience), BV605-conjugated anti-CD64 (BioLegend), PE-conjugated anti-TLR4, and Zombie NIR^™^ live death discriminator (BioLegend) for 15 minutes. 50000 CD64+, F4/80+, TLR4+ (= TLR4 pos. fraction) and CD64+, F4/80+, TLR4- (TLR4 neg. fraction) cells were sorted on an Aria cell sorter from BD and cells were lysed in 350μl RLT buffer (Life Technologies) supplemented with DTT (Sigma) for RNA isolation.

### Western blot

After stimulation, cells were washed with PBS and lysed with M-PER buffer for whole cell lysates or processed according to the instructions of the Nuclear Extraction Kit (Active Motif) to obtain nuclear fractions. An aliquot of each lysate was mixed with NuPAGE^®^ 4x LDS Sample Buffer (Invitrogen) and 50 mM dithiothreitol (Sigma-Aldrich) and boiled for 5 min at 95°C. Proteins were separated by SDS-polyacrylamide gel electrophoresis and transferred onto nitrocellulose membranes (Invitrogen). Membranes were subsequently blocked with 3% BSA in water for 30 min and incubated with the primary antibody overnight at 4°C (Table 1). After washing three times with TBST, secondary antibodies were diluted in 3% BSA in TBST and incubated for 1 h at room temperature. Unbound antibodies were removed by three washing steps and ECL solution was added to membranes for chemiluminescent detection. Densitometric analysis was performed with Optiquant software (Packard Instrument Co., Meriden CT, USA).

**Table 1:** Primary antibodies used in Western blot and the dilution used for each one.

Primary antibody	Dilution
Gp96 (Enzo Life Sciences)	1:2000
p-IκBα (Cell Signaling)	1:1000
IκBα (Cell Signaling)	1:1000
p-NFκB (Cell Signaling)	1:1000
Lamin A/C (Cell Signaling)	1:2500
p-ERK (Cell Signaling)	1:1000
ERK (Cell Signaling)	1:1000
p-p38 (Cell Signaling)	1:1000
p38 (Cell Signaling)	1:1000
β-actin (Millipore)	1:5000

### RNA extraction and real time quantitative PCR

Total RNA was extracted from MM6 cells and peritoneal macrophages using the RNeasy Mini Kit and the automated sample preparation system QIAcube following manufacturer’s instructions (Qiagen, Hilden, Germany). Reverse transcription was performed using the High Capacity cDNA Reverse Transcription Kit (Applied Biosystems, Foster City, CA, USA). Gene expression was determined with TaqMan^®^ Gene Expression Assays Hs00174103_m1 (human IL-8), Mm04208136_m1 (mouse IL-8), Mm00446190_m1 (mouse IL-6), Mm99999068_m1 (mouse TNF-α), Mm01253173_m1 (mouse gp96) (Applied Biosystems). In all cases β-actin was used as endogenous control. Samples were analyzed as triplicates and calculation of relative mRNA expression was performed by ΔΔCt method.

### Detection of cytokine secretion

ELISA directed against IL-8 was conducted with cell culture supernatant using human IL-8 ELISA Kit (Life Technologies) according to manufacturer’s instructions.

### Statistical analysis

Statistical analysis was performed using One-way ANOVA followed by a Newman-Keuls Multiple Comparison Test using the software GraphPad Prism 5. Data are expressed as mean ± S.E.M. Comparison of two groups was performed using Student’s *t*-test. P-values < 0.05 were considered significant.

## Results

### Lentiviral knockdown of gp96 in MonoMac 6 (MM6) cells

**Fig 1:**
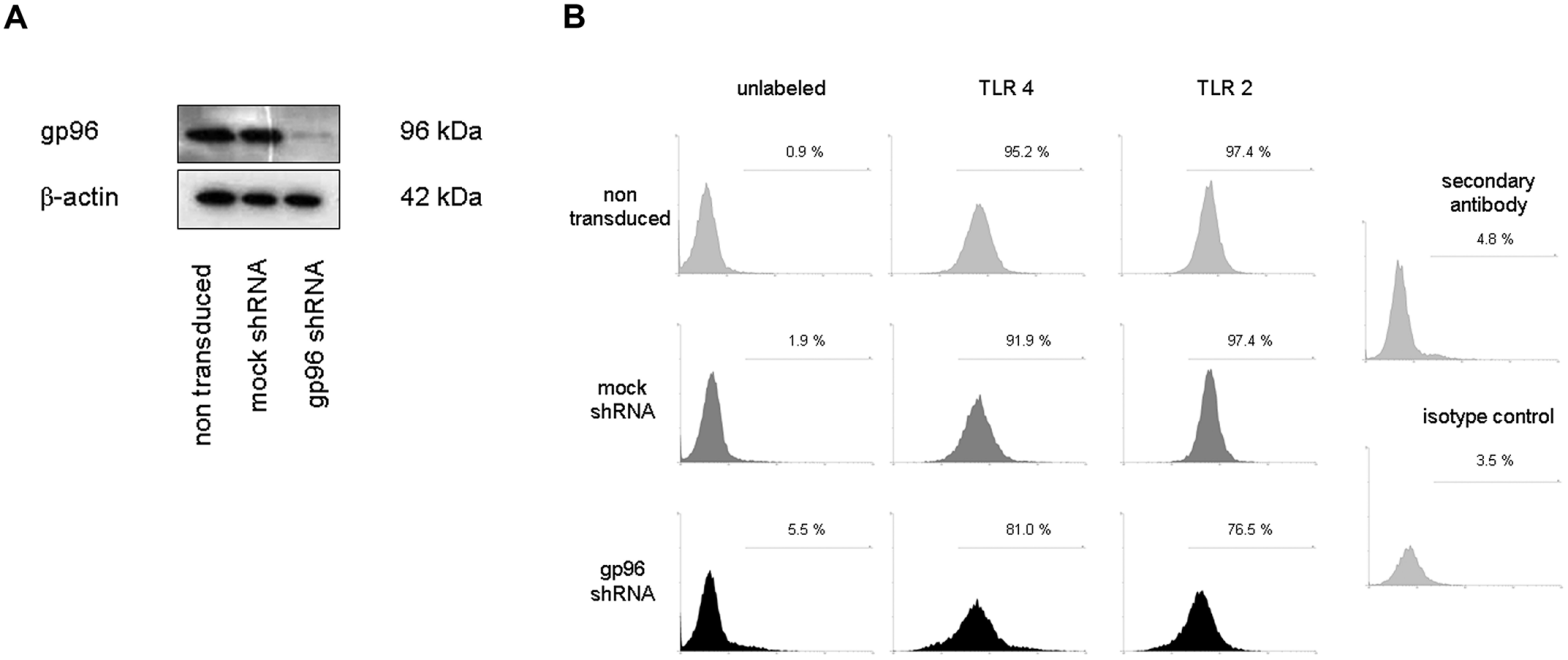
Analysis of TLR 2 and 4 receptors in transduced MM6 cells. A) For stable knockdown of gp96 in MM6 cells a lentiviral system was used and shRNA-expressing cells selected using puromycin. Efficiency of the lentiviral knockdown was more than 90% as determined by Western blot and densitometric analysis. B) Non-transduced, mock shRNA transduced, as well as gp96 shRNA transduced MM6 cells were incubated with antibodies against TLR 4 (second column) and TLR 2 (third column). Subsequent flow cytometric analysis revealed comparable expression of TLR4 and TLR2 on the cell surface of gp96-knockdown MM6 cells (black) and non-transduced (light gray), as well as mock shRNA MM6 cells (dark gray). Given numbers indicate the relative amounts of TLR 2 and 4 positive cells. Secondary antibody and isotype control served as negative controls (fourth column).

To investigate whether missfolding and resulting malfunction of TLRs might be the underlying mechanism by which loss of gp96 in IMAC of CD patients contributes to the observed loss of tolerance against the host gut flora, we generated a stable gp96-knockdown in the human monocytic cell line MM6. For this purpose, lentiviral vector particles were produced in HEK 293T cells, using a vector carrying either a gp96 specific shRNA or a non-targeting shRNA. The latter does not target any human gene, as its short-hairpin sequence contains 5 base pair mismatches to any known human sequence (hereinafter referred to as mock shRNA). As shown in Fig 1A knockdown of gp96 was monitored by Western blot and efficiency of the lentiviral knockdown was more than 90% as determined by densitometric analysis. Transduction with the non-targeting shRNA mock-vector did not affect gp96 protein expression and neither the mock- nor the gp96- specific vector exerted any unspecific effects on basal protein expression levels in MM6 cells.

### Cell surface expression of TLR2 and TLR4 expression is diminished but still present following gp96-knockdown in human monocytes/macrophages

In order to test the hypothesis that gp96 is the major chaperone for TLR2 and TLR4 in human MM6 cells, we investigated the surface expression pattern of these TLRs on non-transduced, mock shRNA- and gp96 shRNA-transduced cells by flow cytometry. If gp96 would be absolutely crucial for TLR surface expression it would be expected that gp96 shRNA transduced cells show a significantly different expression of TLR 2 and/or 4, since un- or misfolded TLRs would not be transported to the cell surface. Cells were incubated with primary antibodies against TLR2 coupled to PE and TLR4 with a secondary PE-labelled antibody. The amount of positively stained single cells versus unstained single cells was assessed. As shown in Fig 1B 95% and 92% of non-transduced (light gray) and mock-transduced MM6 cells (dark grey) were positive for TLR4 (second column). Analysis of gp96 shRNA transduced cells (black) revealed a decrease, as a portion of 81% was found to be positive. According results were obtained for TLR2 expression (third column). 97% of non- and mock-transduced cells were found to be positive, whereas 77% of gp96 shRNA transduced cells were positive for TLR2. These results indicate that loss of gp96 leads to a decreased, however still substantial expression of TLR2 and TLR4 on the cell surface. We cannot exclude that less than 10% of remaining gp96 expression is still sufficient to facilitate some TLR folding and surface expression. Therefore, we performed further experiments to better clarify this aspect.

### Activation of IκBα and NF-κB upon stimulation with LPS is not significantly impaired upon gp96-knockdown

**Fig 2:**
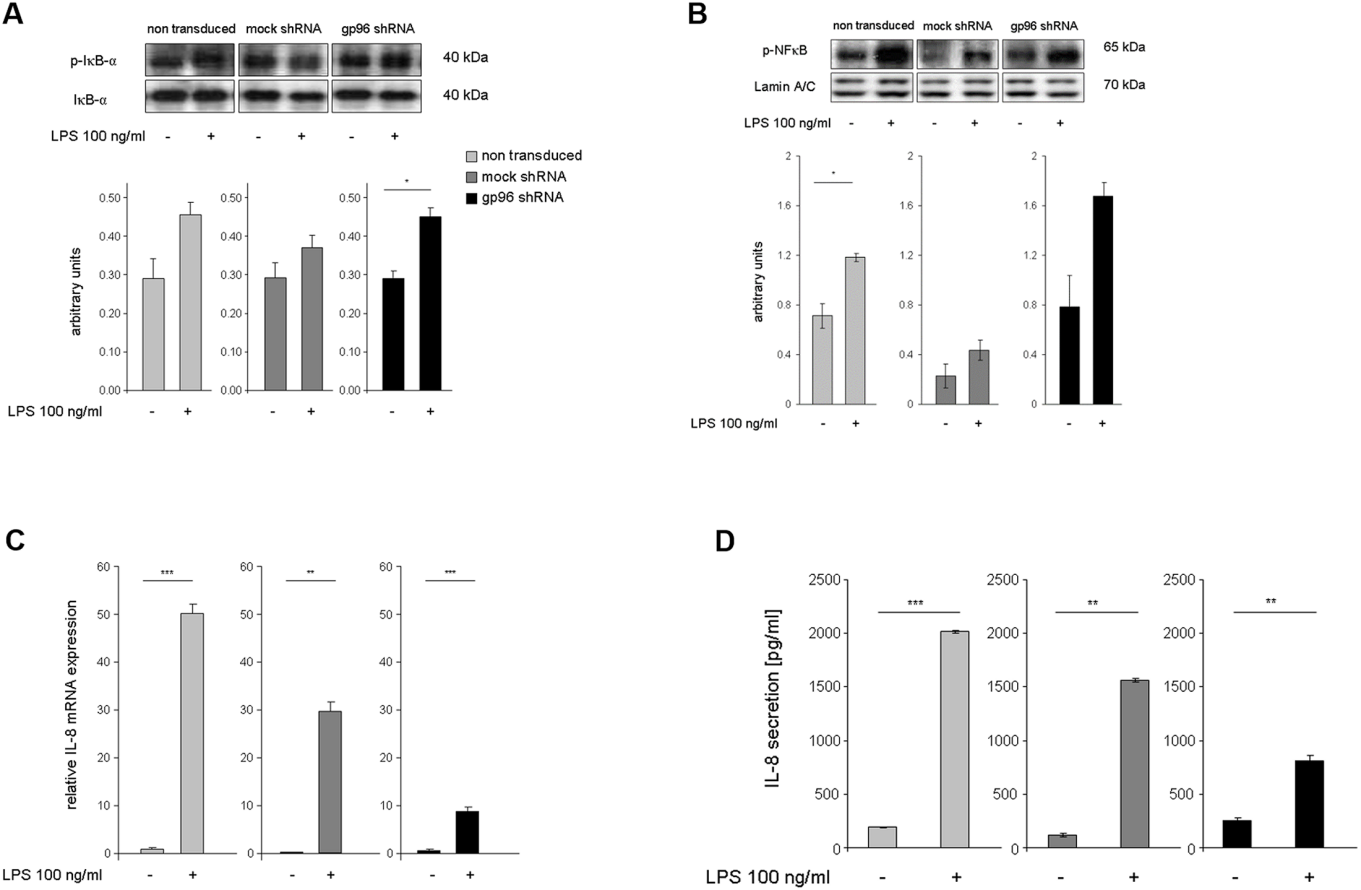
LPS stimulation activates NF-κB signalling and the expression of IL-8 in gp96 shRNA transduced MM6 cells. Non-transduced, mock shRNA transduced and gp96 shRNA transduced MM6 cells were treated with LPS (100 ng/ml) for 2 h. A) Western blot and subsequent densitometric analysis of whole cell lysates revealed unimpaired phosphorylation of IκB-α, the inhibitor of NF-κB, upon stimulation with LPS under all three conditions. The presented Western blots are representative for 3 independent experiments. Densitometric analysis below represents the ratio between p-IκB-α and IκB-α (n = 3). Data are represented as mean +/- SEM. Statistical analysis was performed using Student’s *t*-test. Asterisks denote significant differences from the respective control. * = *P*<0.05. B) Western blot and subsequent densitometric analysis of nuclear fractions revealed relocation and accumulation of p-NF-κB into the nucleus upon stimulation with LPS for 2 h under all three conditions. Lamin A/C served as loading control. The presented Western blots are representative for 3 independent experiments. Data are represented as mean +/- SEM. Statistical analysis was performed by Student’s t-test. Asterisks denote significant differences from the respective control (* = *P*<0.05). C) mRNA expression of IL-8 (n = 3) after 2 h relative to housekeeping gene β-actin. D) Secretion of IL-8 protein into the cell culture supernatant after 24 h of stimulation as determined by ELISA. Data are represented as mean +/- SEM. Statistical analysis was performed using Student’s t-test. Asterisks denote significant differences from the respective control. * = *P*<0.05, ** = *P*<0.01, *** = *P*<0.001.

Subsequently, the functionality of TLR 4 in gp96-knockdown cells was investigated. For this purpose, cells were treated with LPS (100 ng/ml) for 2 h. Functionality was analyzed by analyzing the activation of the NF-κB signalling pathway assessing the phosphorylation of NF-κB and IκBα, the inhibitor of NF-κB, by Western blot. As shown in Fig 2A, Western blot and subsequent densitometric analysis of whole cell lysates revealed unchanged phosphorylation of IκBα upon stimulation with LPS under all three conditions. Densitometric analysis of the ratio between p-IκBα and IκBα (n = 3 each) showed increasing amounts of p-IκBα upon stimulation with LPS. For non-transduced MM6 cells ratios increased 1.6-fold from 0.29±0.05 to 0.46±0.03. For mock shRNA cells a 1.3-fold increase from 0.29±0.04 to 0.37±0.03 was found. For gp96 shRNA cells a significant 1.6-fold increase from 0.29±0.02 to 0.45±0.02 was determined.

Western blot of nuclear lysates (Fig 2B) revealed translocation and accumulation of p-NF-κB in the nucleus upon stimulation with LPS for 2 h under all three conditions. Densitometric analysis of the ratio between p-NF-κB and NF-κB (n = 3 each) showed increasing amounts of p-NF-κB upon stimulation with LPS. For non-transduced MM6 cells ratios increased 1.6-fold from 0.71±0.1 to 1.18±0.03. For mock shRNA cells a 1.9-fold increase from 0.23±0.1 to 0.44±0.08 was found. For gp96 shRNA cells also a 2.1-fold increase from 0.78±0.26 to 1.67±0.11 was determined. These results indicate that gp96-knockdown does not significantly alter activation of signal transduction via TLR4 in human macrophages.

### Interleukin (IL)-8 expression and secretion is induced but to a reduced amount in gp96-knockdown cells upon stimulation with LPS

In order to investigate whether the activation of the NF-κB signalling pathway leads to increased transcription of typical target genes, IL-8 expression and secretion were analyzed after treatment of either non-transduced MM6, mock shRNA or gp96 shRNA transduced MM6 cells with LPS (100 ng/ml) for 2 h and 24 h, respectively. Fig 2C shows the mRNA expression of IL-8 (n = 3) after an incubation of 2 hours relative to the housekeeping gene β-actin. IL-8 is strongly upregulated (60-150-fold) in non-transduced (light gray, 0.85±0.33 to 50.3±1.9) and mock shRNA cells (dark gray, 0.18±0.19 to 29.5±2.1). In gp96 shRNA (black) transduced cells a 20-fold increase of IL-8 mRNA was found (0.43±0.22 to 8.59±1.0).

As shown in Fig 2D, secretion of IL-8 into the cell culture supernatant after 24 h of stimulation correlates with changes in IL-8 mRNA expression. IL-8 secretion was strongly increased (11 and 13-fold, respectively) in non-transduced (light gray, 191±3 pg/ml to 2015±5 pg/ml) and mock shRNA cells (dark gray, 118±2 pg/ml to 1562±11 pg/ml). In gp96 shRNA transduced cells (black) a 3-fold increase of IL-8 secretion was found (254±12 pg/ml to 808±40 pg/ml).

LPS-mediated IL-8 expression as well as its subsequent secretion by gp96-knockdown cells was reduced compared to that of control cells. However, it was still significantly increased.

### Generation of the conditional LysMCre-gp96floxed-mice

**Fig 3:**
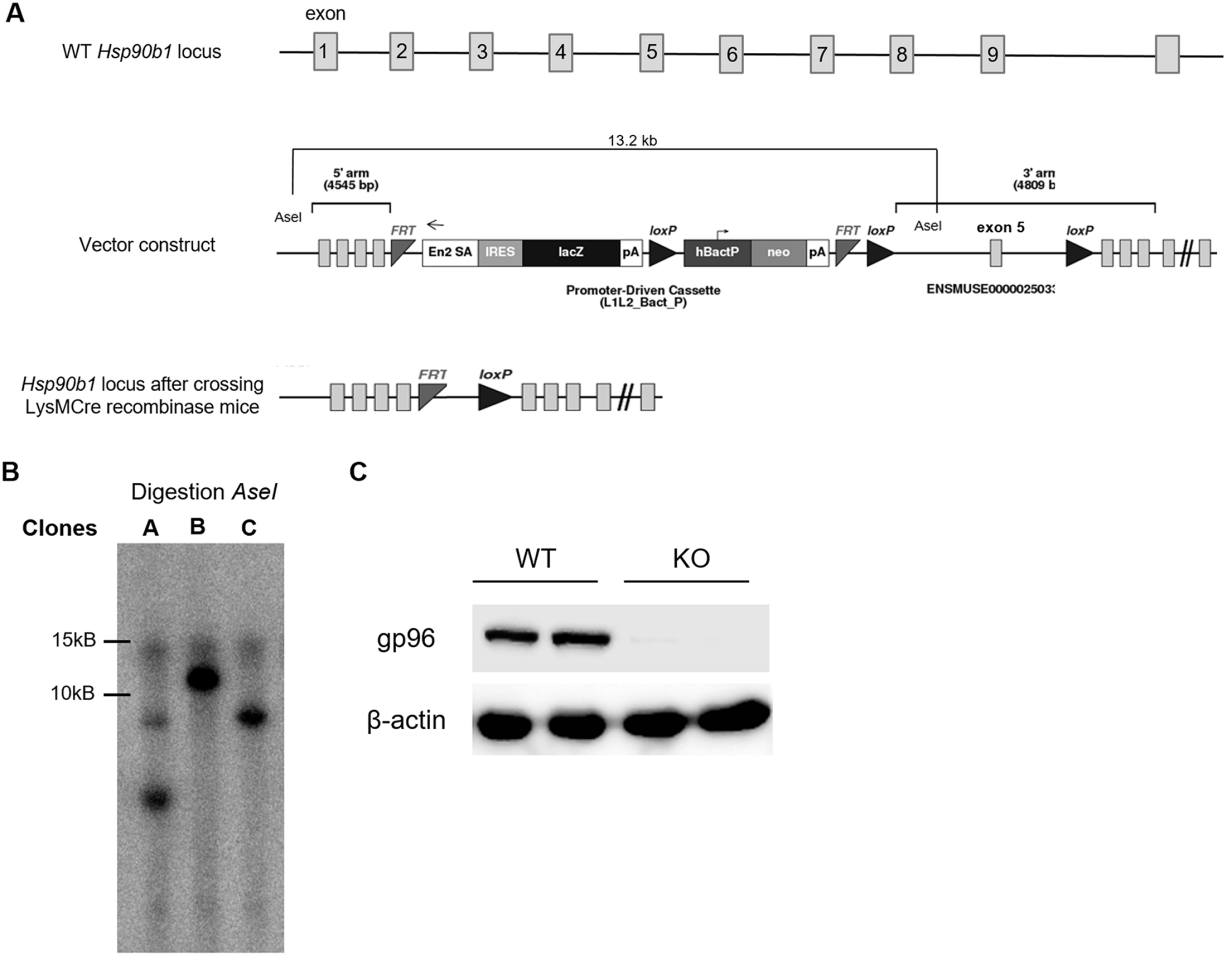
Generation of conditional LysMcre-gp96floxe-mice. A) Scheme of the Hsp90b1 locus, the vector that was generated and the resulting locus after each breeding step. B) Southern Blot using an internal probe in the Neo sequence to test ES clones. Representative agarose gel showing three different clones after AseI digestion. Clone B was selected as the only clone with correct insertion of the construct as it was the only one with the correct size of the fragment after AseI digestion (13.2Kb) C) Peritoneal macrophages from both WT and conditional KO mice were isolated and Western blot analysis for gp96 was performed. The picture shows a representative Western Blot from peritoneal macrophages and each line represents one mouse.

As we had found a reduction but not absence of pro-inflammatory signaling and gene expression upon TLR stimulation in gp96 deficient cells we further aimed to generate supporting *in vivo* data. For this purpose, we generated a myeloid-specific gp96-knock-out mouse using a construct from EUCOMM with the gp-96 exon 5 flanked by LoxP sequences (Fig 3A). As described in the experimental procedures section, we generated the macrophage-specific gp96-KO mice after crossing with FLP mice in order to remove the gene-trap cassette and with LysMCre mice to obtain a knock-out of gp96 specifically in myeloid cells. Southern blot was performed in order to verify the correct size and select the correct clone. As shown in Fig 3B, clone B was selected since it was the clone which yielded a fragment of 13.2Kb after AseI digestion. Western blots on peritoneal macrophages isolated from Hsp90b1-flox (WT) and LysMcre-Hsp90b1-flox (gp96-KO) mice (Fig 3C) indicated absence of gp96 protein in macrophages from gp96-KO mice.

### TLR2 is still present in macrophages from g96-KO mice, whereas TLR4 is absent

**Fig 4:**
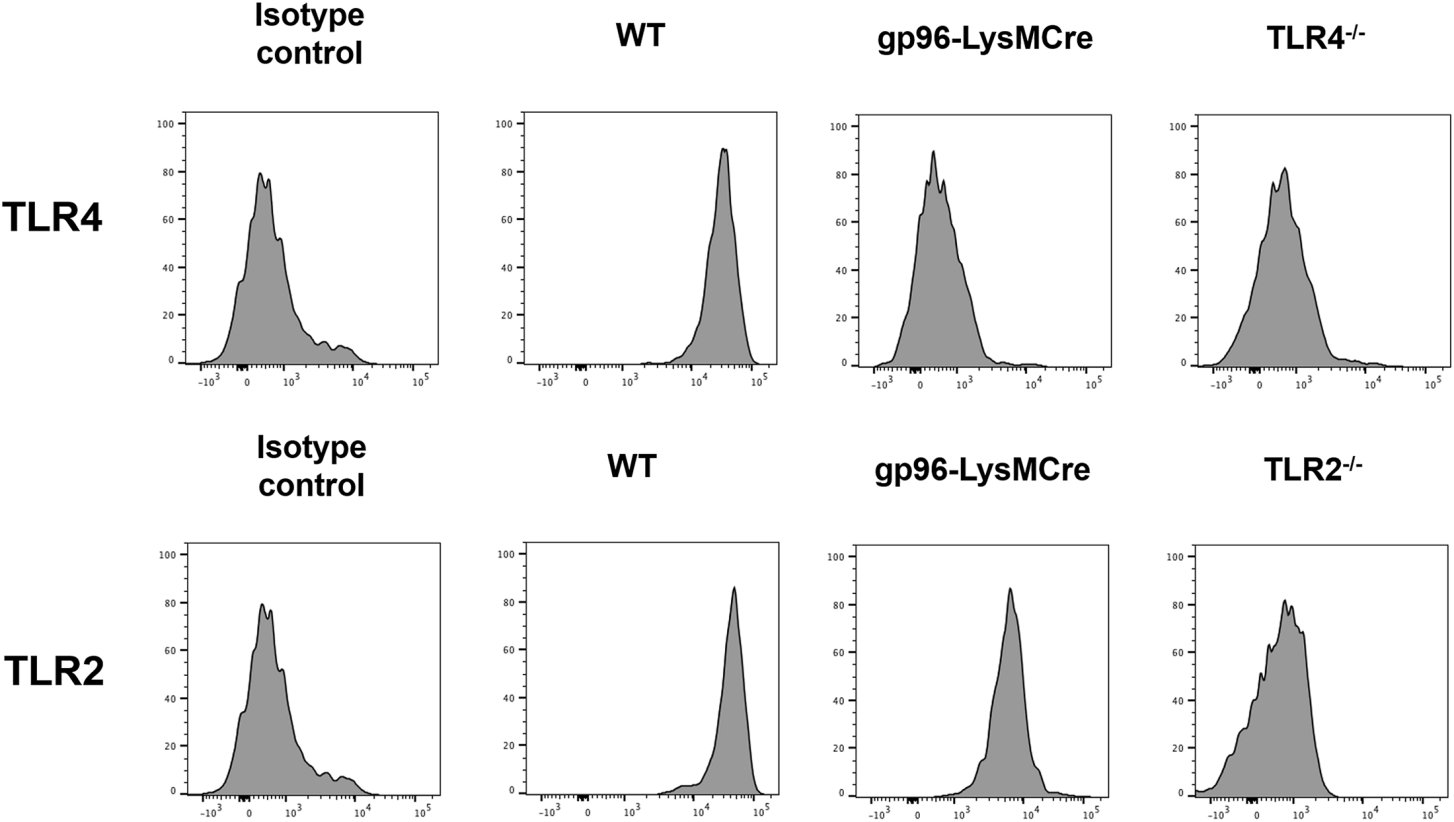
Analysis of TLR2 and TLR4 surface expression. Peritoneal macrophages were isolated from WT, conditional gp96-KO, Tlr2-KO and Tlr4-KO mice and the expression of the surface receptors TLR2 and TLR4 was performed by flow cytometry. The histograms are representative for one out of three independent experiments.

After generation of the LysMcre-gp96-flox mice, peritoneal macrophages from: WT, conditional gp96-KO, Tlr2-/- and Tlr4-/- mice were isolated and flow cytometry analysis for TLR2 and TLR4 expression was performed. The purity of the peritoneal macrophages was higher than 97% (S1 Fig). As shown in Fig 4, surface TLR4 expression was absent on macrophages from gp96-KO mice and there was no difference regarding TLR4 expression between macrophages from gp96-KO mice and those from Tlr4-/- mice. The analysis of TLR2 receptor revealed a slight decrease in its surface expression level on macrophages from gp96-KO mice compared to those obtained from WT mice, but a significant increase compared with those obtained from Tlr2-/- mice (Fig 4). These results supported the cell line data indicating that absence of gp96 causes a reduction of cell surface TLR2 but not the complete absence of Tlr4 receptor observed in peritoneal macrophages and not in gp96 shRNA MM6 cells.

### LPS-TLR4 mediated induction of NF-κB signaling and cytokine production is still preserved in macrophages from LysMcre-gp96-flox mice

**Fig 5:**
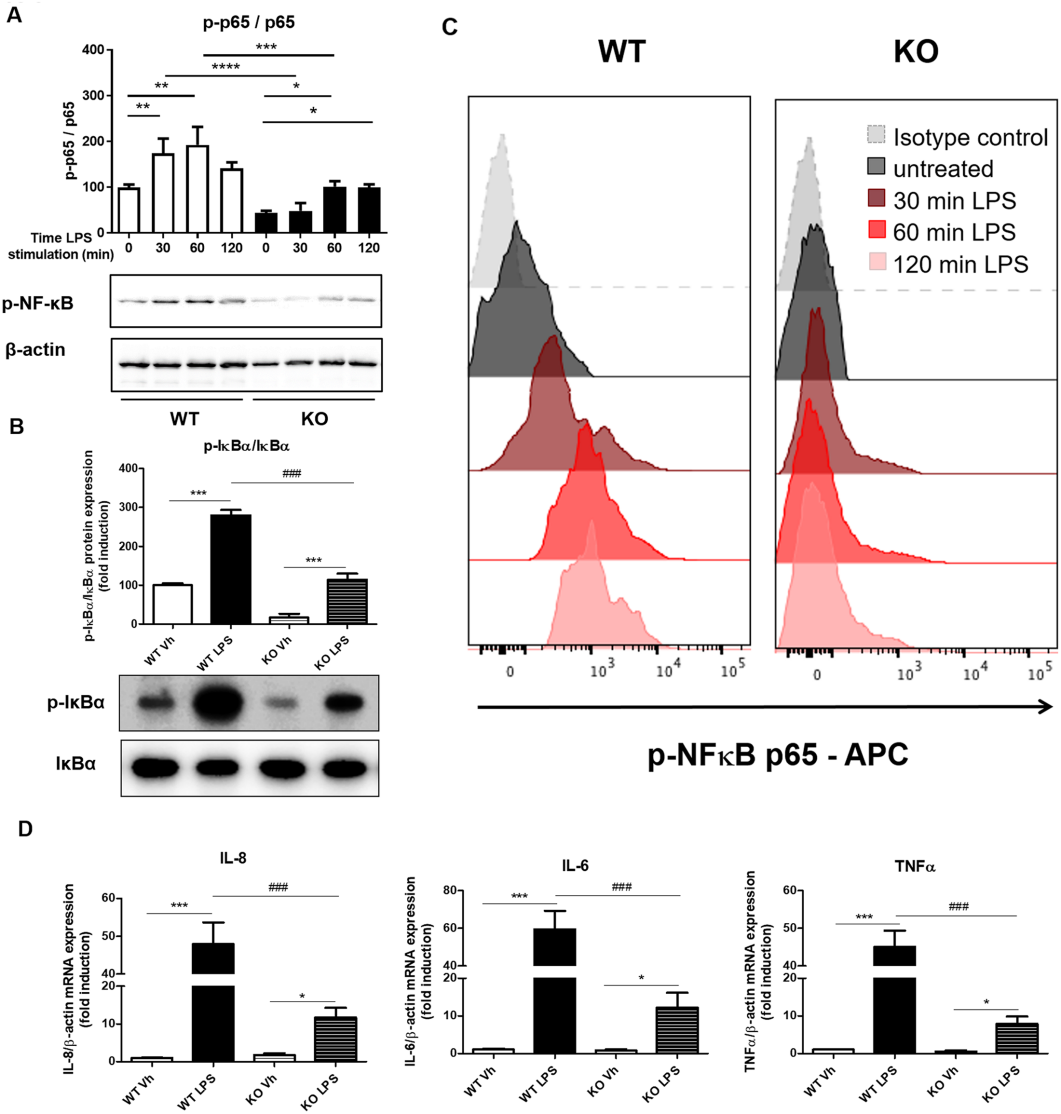
LPS-TLR4 signalling is active in both WT and conditional LysMcre-gp96flox-mice. Peritoneal macrophages were isolated from both WT and conditional gp96-KO and treated with lipopolysaccharides (LPS) (100ng/ml) for 30, 60 and 120 minutes. A and B) Total protein was isolated after LPS treatment and Western blots performed for p-IκBα, IκBα, p-NF-κB and β-actin. Graphs show the densitometry quantification of the blots (n = 3). C) After LPS treatment, macrophages from both WT and conditional gp96-KO were permeabilized and the expression of p- NF-κB was analysed by flow cytometry. The histograms are representative of three independent experiments. D) RNA was isolated after LPS treatment and the expression of IL-6, IL-8 and TNF-α mRNA was measured by real-time quantitative PCR. “Vh” represents macrophage treated with vehicle. Graphs show the fold induction of each gene (n = 4). In all cases data are expressed as mean±SEM. Statistical analysis was performed by One-way ANOVA followed by a Newman-Keuls test. * = *P*<0.05, *** = *P*<0.001 vs respective non-treated macrophages and ### = *P*<0.001 vs LPS-treated macrophages from WT mice.

Following our results in MM6 cells upon gp96 silencing, we sought to investigate whether TLR4 is still functional under gp96-KO conditions. Hence, we isolated peritoneal macrophages from both WT and gp96-KO mice, and treated them with LPS for different times (0, 30min, 60 min, 120min and 24 hours) to activate TLR4 and analyze downstream signalling. Protein levels ofp-NF-κB, p-IκBα and subsequent mRNA expression levels of IL-8, IL-6 and TNF-α were analyzed. The treatment with LPS induced a significant increase in the ratio of p-NF-κB / NF-κB at 30, 60 and 120 minutes in macrophages from WT mice (174±18.6, 192.9±22.7 and 141.8±7.4 respectively). Without LPS treatment, peritoneal macrophages from gp96-KO mice exhibited lower levels of the ratio p-NF-κB / NF-κB (44.24±2.5), but treatment with LPS significantly increased this ratio after 60 minutes (101.7±6.8) and 120 minutes (99.9±3.9) when compared to non-treated macrophages. (Fig 5A). In line with this, at 24 hours of LPS treatment, the ratio of p-IκBα /IκBα revealed a significant increase in macrophages from bothWT (280.0±13.4%) and gp96-KO (114.0±15.2%) mice when compared to the respective non-treated macrophages (100.0±4.7% and 16.6±9.9%, respectively, Fig 5B). These results were strongly reinforced with FACS experiments that revealed a significant increase in the number of p-p65+ macrophages after treatment with LPS in a time-dependent manner, which peaked at 30 minutes, in macrophages from both WT and gp96-KO mice (Fig 5C).

The expression of pro-inflammatory cytokines such as IL-8, IL-6 and TNF-α was significantly induced of in macrophages from both WT (47.9±5.7, 59.4±9.9 and 45.1±4.3 fold induction, respectively) and KO (11.7±2.6, 12.3±3.9 and 7.9±1.9 fold induction, respectively) mice upon LPS stimulation (Fig 5D). However, LPS induced a significantly lower expression of these pro-inflammatory cytokines in conditional gp96-KO mice, as compared to WT mice supporting the cell line data.

### Tlr4 expression is induced in gp96-deficient macrophages upon LPS-treatment

**Fig 6:**
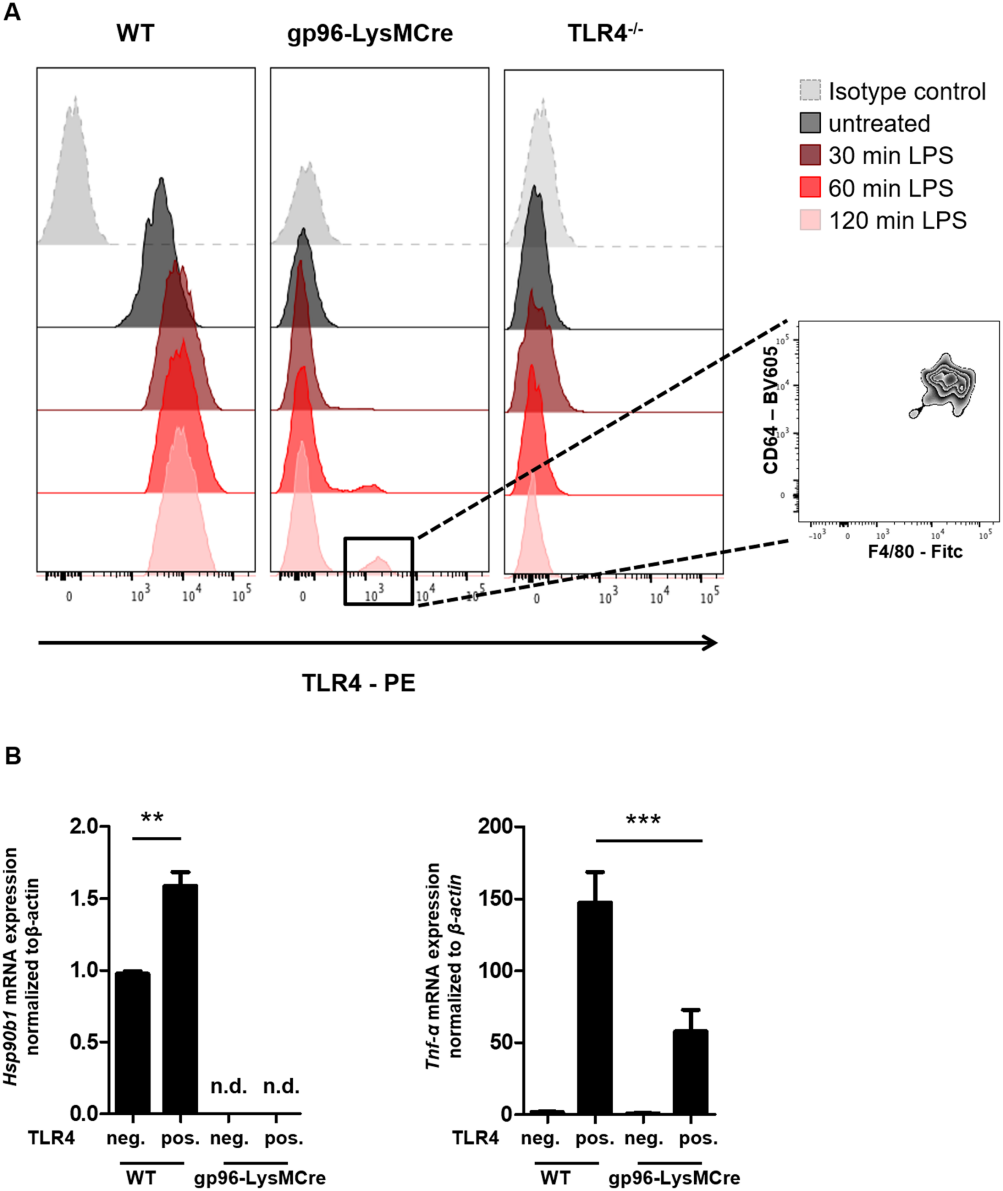
LPS stimulation induces the expression of TLR4 in gp96-deficient macrophages. A) Peritoneal macrophages from WT, conditional gp96-KO and Tlr4-KO mice were treated with LPS for 30, 60 and 120 minutes and the surface expression of TLR4 was analysed by flow cytometry. The histograms are representative for one out of three independent experiments. B) Peritoneal macrophages from WT and gp96-KO mice were treated with LPS for 120 minutes and CD64+, F4/80+, TLR4+ (pos.) and CD64+, F4/80+, TLR4- (neg.) macrophages sorted by flow cytometry. RNA from those cells was obtained and the expression of gp96 and TNFα was analysed by real-time quantitative PCR. Graphs show the amount of indicated genes relative to TLR4- macrophages from WT mice (n = 3). In all cases data are expressed as mean±SEM. Statistical analysis was performed by One-way ANOVA followed by a Newman-Keuls test. ** = *P*<0.01, *** = *P*<0.001.

Since TLR4 is the main receptor sensing LPS, we next assessed why macrophages from gp96-KO mice were able to react to LPS treatment. In order to clarify this discrepancy, we analyzed the expression of TLR4 receptor after treatment with LPS in macrophages from gp96-KO mice. As expected, LPS-treatment induced a significant increase in TLR4 expression in WT macrophages. Of interest, however, we also found a significant induction of surface TLR4 expression on a portion of macrophages from gp96-KO mice in a time-dependent manner. The TLR4+ population clearly consisted of macrophages, since these cells expressed high levels of the macrophage markers CD64 and F4/80 (Fig 6A).

To confirm that those cells do not express gp96 and are not a small fraction of remaining gp96-positive cells, we sorted these TLR4+ cells and analyze the expression of gp96 mRNA in both TLR4- and TLR4+ cells. As a control, we also analyzed the expression of gp96 in TLR4+ and TLR4- cells from WT mice. We did not detect gp96 in TLR4+ or TLR4- macrophages isolated from gp96-KO mice, again confirming that gp96 is dispensable for TLR4 induction (Fig 6B). Interestingly, TLR4+ macrophages from WT mice exhibited significantly higher levels of gp96 mRNA than TLR4- macrophages (Fig 6B). Of interest, the expression of TNF-α was significantly lower in TLR4+ cells from gp96-KO mice compared with TLR4+ cells from WT mice (Fig 6B), which reinforces the fact that gp96 is involved in regulating signaling cascades downstream of TLR4.

### Gp96 deficiency reduces the function of TLR4 and M-CSF receptor via reduction of phosphorylated kinases

**Fig 7:**
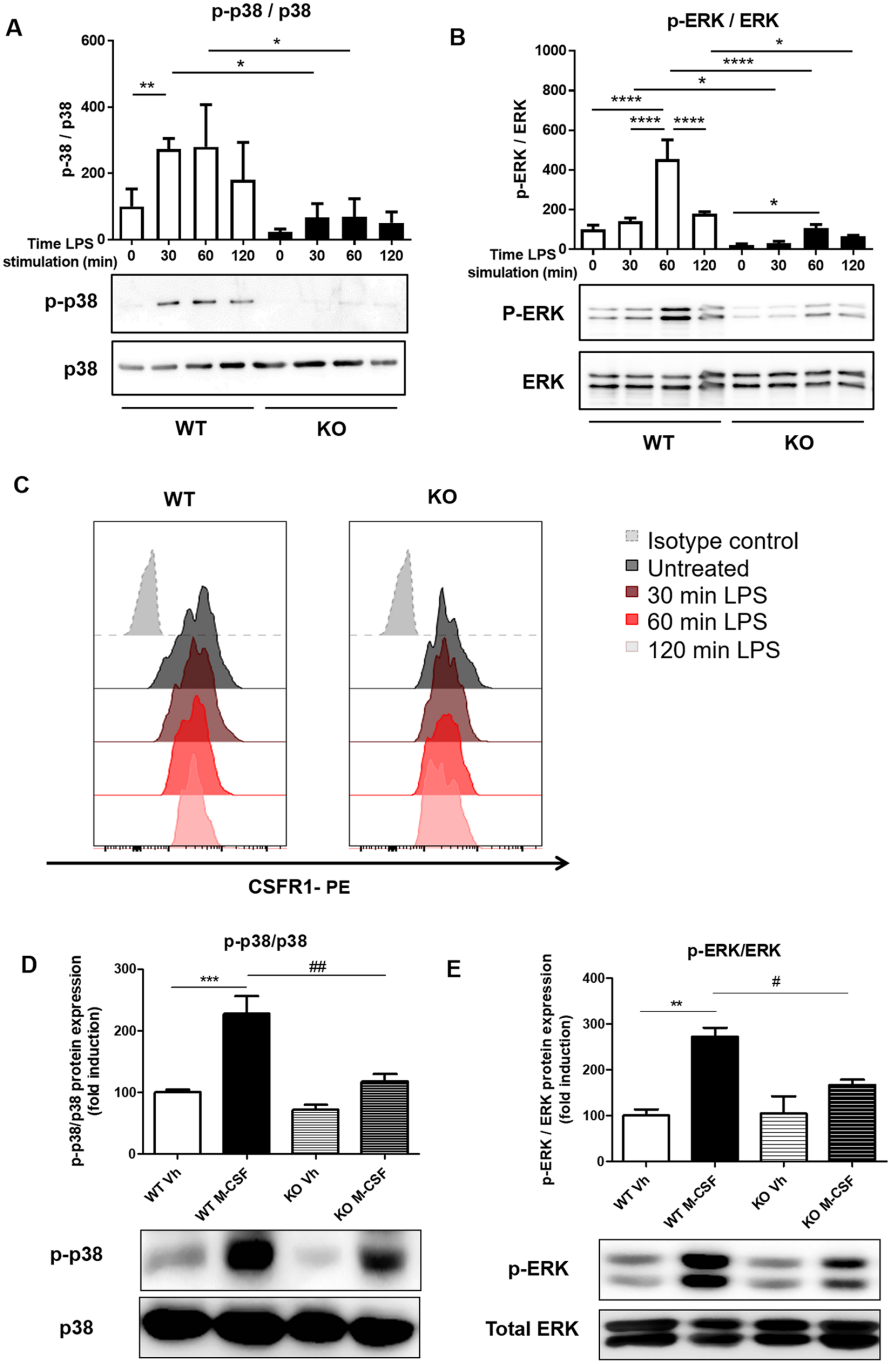
Gp96 deficiency induces an impaired phosphorylation of ERK and p38. A and B) Peritoneal macrophages were isolated from both WT and conditional KO mice and treated with lipopolysaccharides (LPS) (100 ng/ml) for 30, 60 and 120 minutes. C) The surface expression of CSF1R was analysed by flow cytometry in CD64+, F4/80+ peritoneal macrophages from both WT and conditional gp96-KO mice. The histograms are representative for one out of three independent experiments. D and E) Peritoneal macrophages were isolated from both WT and conditional KO mice, and treated with 10 ng/ml Macrophage Colony-stimulating Factor (M-CSF) for 3 days and restimulated with 10 ng/ml M-CSF for 10 minutes. Total protein was then isolated and Western blots for p-ERK, ERK, p-p38 and p38 were performed. “Vh” stands for macrophage treated with vehicle. Graphs show densitometric quantification of the blots (n = 5). In all cases data are expressed as mean±SEM. Statistical analysis was performed by One-way ANOVA followed by a Newman-Keuls test. ** = *P*<0.01, *** = *P*<0.001 vs respective non-treated macrophages and # = *P*<0.05, ## = *P*<0.01 vs M-CSF treated macrophages from WT mice.

Since TLR4 was still–at least partial—functional in LysMCre gp96 KO mice, we further analyzed other kinases involved in downstream signaling pathway. LPS treated peritoneal macrophages from WT mice showed a significant increase in p-p38 at 30 minutes (273.4±18.7) and an increase, although not significant, at 60 minutes (280.1±73.5) and 120 minutes (180.6±65.3). Moreover, the ratio of p-ERK/ERK showed a significant increase at 60 minutes (454.8±56.4) and at 120 minutes (192.3±9.7) (Fig 7A and 7B). On the other hand, macrophages from KO mice exhibited significantly lower levels of p-p38 (22.72±5.5) and p-ERK (20.73±4.2) compared with macrophages from WT mice. However, LPS treatment induced a significant increase of p-ERK only at 60 minutes (107.9±10.2) compared with non-treated macrophages, whereas only a slight and non-significant increase was detected in the amount of p-p38 (Fig 7A and 7B).

In order to determine whether the reduction of p-p38 and p-ERK was only linked to TLR4 receptor or also another receptor, we analyzed the Colony stimulating factor 1 receptor (CSF1R), another receptor, which also promotes the phosphorylation of p38 and ERK. First, we analyzed the expression of CSF1R, to address whether gp96 might be involved in surface expression of this receptor, but no significant differences were observed between macrophages from WT compared with macrophages from KO (Fig 7C). Next, peritoneal macrophages from both WT and KO mice were treated with M-CSF and, as shown in Fig 7D and 7E, results revealed a significant increase of p38 and ERK phosphorylation in peritoneal macrophages from WT (227.4±29.0% and 271.6±20.1%, respectively), but not macrophages from KO mice (117.0±12.4% and 166.8±11.2%, respectively) upon treatment with M-CSF.

## Discussion

The present study demonstrates that TLR2 is decreased but still present in macrophages under gp96 knock down conditions. Further, although TLR4 was absent from the surface of gp96-deficient macrophages, these cells were still able to induce TLR4 expression upon LPS treatment. However, signaling via TLRs was found to be impaired due to a decreased stress kinase phosphorylation. In contrast, signaling via NF-kB was only slightly affected upon loss of gp96.

It has been suggested that monocyte/macrophage-specific deletion of gp96 causes a loss of TLR2 and TLR4 surface expression leading to a conditional and cell-specific TLR null mouse [5]. TLRs represent key mediators of innate host defense in the intestine, involved in maintaining mucosal as well as commensal homeostasis [21]. Under healthy conditions, TLR signalling drives essential mechanisms for protecting host barrier integrity and maintaining commensal composition and tolerance. However, aberrant or dysfunctional TLR signalling may impair commensal-mucosal homeostasis. Therefore, it contributes to the onset and perpetuation of tissue injury and consequently leads to chronic inflammation in IBD [21]. Here we provide evidence that gp96 indeed is a chaperone for TLRs, but a lack of gp96 does not cause a complete loss of TLR2, and only under basal conditions leads to a complete loss of TLR4, while upon LPS stimulation TLR4 can still be induced. Hence, the lack of gp96 does not induce a TLR2 and TLR4 null phenotype.

In contrast to other reports, the results obtained in MM6 cells showed a reduction, but not an abolishment, of TLR surface expression. Indeed, the functionality of TLR2 and TLR4 upon stimulation with LPS was partially maintained as indicated by normal phosphorylation of IκBα as well as unchanged translocation and accumulation of p-NF-κB in the nucleus. The expression, as well as the subsequent secretion of IL-8, a typical target gene of activated NF-κB, was reduced by gp96-knockdown but was still significantly increased upon LPS stimulation. While experiments with peritoneal macrophages from our conditional gp96 knock-out mice confirmed retained expression of TLR2 in gp96-deficient macrophages, TLR4 was absent at basal conditions in those cells. This apparent discrepancy could be explained by the fact that shRNA mediated knockdown of gp96 might be only partial in MM6 cells, so the remaining amount of gp96 present in gp96 shRNA could be responsible for presence of TLR4 receptor and functional response to LPS treatment.

Analysis of TLR4 receptor revealed that macrophages from our conditional gp96-KO mice do not express this receptor at the surface in absence of stress signals. These results perfectly fit with numerous previous observations which described the important role of gp96 in the folding of TLRs receptors in immune cells such as macrophages and B-cells [22–24], and also with the previous work of another group which generated a different conditional LysMcre gp96-knock-out mouse where they observed that gp96 is the master chaperone of TLR4 receptor [6]. Our data expand these observations showing that, although gp96 is essential for the correct folding of TLR4 receptor under basal condition, LPS is able to induce the expression of TLR4 receptor even in the absence of gp96. As far as we are concerned, this is the first report showing that the expression of TLR4 receptor can be induced after LPS stimulation in the absence of gp96, suggesting that there has to be another chaperone involved in TLR4 induction upon stress responses. These results were strongly reinforced when peritoneal macrophages from both WT and conditional gp96-KO mice were treated with LPS, and an induction of p-NF-κB and the expression of pro-inflammatory cytokines such as IL-8, IL-6 and TNF-α was observed in all cells. Nevertheless, it is important to note that we have deleted the exon 5 of gp96, unlike previous reports that deleted the exon 1[6]. Although we did not observe the presence of a truncated gp96 protein using an antibody against the N-terminal part of gp96, we cannot completely rule out the possibility that deleting exon 5 could might yield a truncated protein with a reduced function. This could explain the induction of TLR4 upon LPS treatment observed in peritoneal macrophages from our conditional knockout mice. Hence, further studies should be performed to reproduce these observations in the mice lacking exon 1 and to better elucidate the molecular mechanisms responsible for the induction of TLR4 receptor in the absence gp96.

The presence of co-chaperones has a direct influence on their function. Indeed, it has been described that gp96 requires the presence of another ER luminal protein, CNPY3, in order to induce the folding of TLR9 [25]. Although not any TLR4-specific co-chaperone for gp96 has been described yet, such a protein is impaired also in our conditional gp96-KO mice (data not shown), suggesting that the lack of gp96 affects also the expression of another co-chaperones required for the folding of TLRs receptors. In line with this, it has been recently reported the interactome of gp96, which reveals a huge number of proteins, among others CNPY3, associated to this chaperone, revealing a critical role to integrate innate immunity, Wnt signaling and organ development[26].

The fact that LPS treatment induces the expression of pro-inflammatory cytokines in gp96-deficient macrophages further suggests that gp96 is not the (only) chaperone involved in the folding and functionality of TLR4. Although TLR4 was induced and functional in gp96 deficient macrophages after LPS stimulation, downstream signaling was clearly reduced. These data are in line with our previous findings, showing a clear expression of TLR 2 and 4 on inflammation-associated IMACs in CD patients [18, 27], in which gp96 expression is strongly reduced or even completely absent in IMACs [19, 28]. Of interest, it has recently been reported that gp96 is essential for CD11c+ cells to preserve the gut homeostasis and to induce regulatory T cells, showing the important role of this chaperone in immune responses [29]. Our present study expands our previous observations and points to an impaired phosphorylation of ERK and p38 kinases upon loss of gp96. This is in line with the finding that the functionality of another receptor namely Colony stimulating factor 1 receptor (CSF1R) was also impaired in gp96 deficient macrophages–although surface expression of CSF1R was not impaired. Similarly, a reduction of the phosphorylation of both, ERK and p38 kinases was observed, confirming the importance of gp96 for the activation of these kinases. In line with our results, it has been reported that the exogenous administration of gp96 induces an increased phosphorylation of ERK [30]. The present report highlights the crucial role of this chaperone for phosphorylation of the MAP kinase pathway kinases, ERK and p38, which also fits with the lethality of the complete gp96 knock-out mice reported in 2007 by Wanderling S. *et al*. [31].

In summary, we demonstrate that the lack of gp96 in both the human monocytic cell line MM6 and in macrophages from LysMcre-gp96 floxed mice neither leads to a complete loss of TLR 2 expression nor to a complete loss of TLR-induced signaling, but is associated with an impaired phosphorylation of ERK and p38. These results reveal for the first time a crucial role for gp96 in the regulation of ERK and p38 kinases, which is independent from the surface expression of ERK/p38-inducing receptors, and thus points to a completely novel and so far unknown role for gp96.

## Supporting information

S1 Fig Purity of peritoneal macrophages before FACS analysis. Peritoneal macrophages from both WT and conditional gp96-KO mice were obtained and, purity of macrophages was analysed by flow cytometry for the expression of the macrophage markers CD64 and F4/80. The dot plots are representative for one out of three independent experiments.
(TIF)
