# Supplementary figures and images for: Gp96 deficiency affects TLR4 functionality and impairs ERK and p38 phosphorylation
Source: PLoS One. 2018 Feb 15;13(2):e0193003. doi: 10.1371/journal.pone.0193003 (PMC5814018; doi:10.1371/journal.pone.0193003)

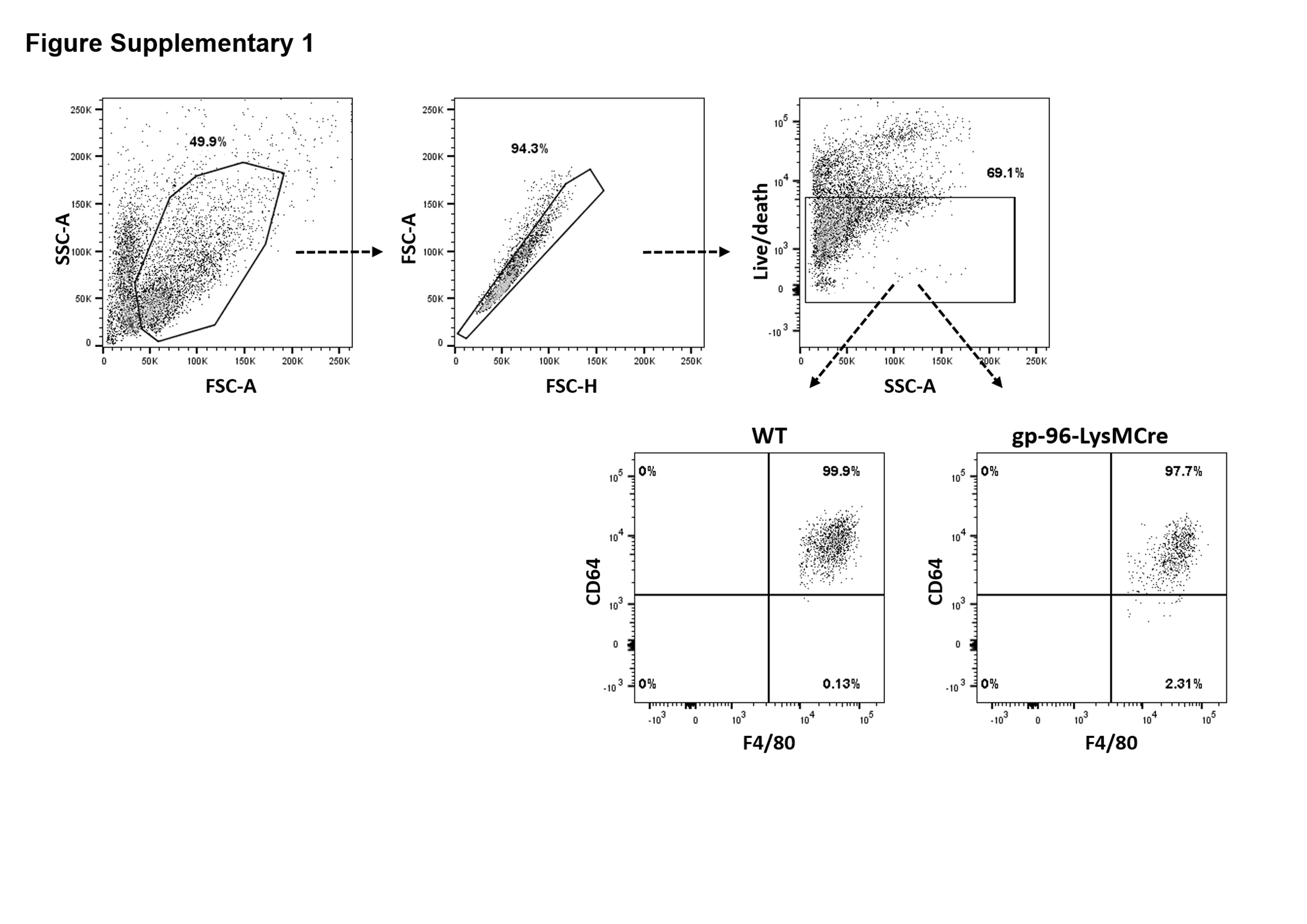

Supplement: S1 Fig — Peritoneal macrophages from both WT and conditional gp96-KO mice were obtained and, purity of macrophages was analysed by flow cytometry for the expression of the macrophage markers CD64 and F4/80. The dot plots are representative for one out of three independent experiments. (TIF) [file pone.0193003.s001.tif]
